# Using artificial intelligence and deep learning to optimise the selection of adult congenital heart disease patients in S-ICD screening

**DOI:** 10.1016/j.ipej.2024.06.003

**Published:** 2024-06-11

**Authors:** Mohamed ElRefai, Mohamed Abouelasaad, Isobel Conibear, Benedict M. Wiles, Anthony J. Dunn, Stefano Coniglio, Alain B. Zemkoho, John Morgan, Paul R. Roberts

**Affiliations:** a Cardiology Department, University Hospital of Cambridge, Cambridge, United Kingdom; b Cardiac Rhythm Management Research Department, University Hospital Southampton NHS Foundation Trust, Southampton, United Kingdom; c Faculty of Medicine, University of Southampton, Southampton, United Kingdom; d Aberdeen Royal Infirmary, Aberdeen, United Kingdom; e School of Mathematical Sciences, University of Southampton, United Kingdom; f Decision Analysis Services Ltd, Basingstoke, United Kingdom; g Department of Economics, University of Bergamo, Italy

**Keywords:** Adult congenital heart disease, S-ICD, Deep learning, Cardiac implantable devices

## Abstract

**Introduction:**

The risk of complications associated with transvenous ICDs make the subcutaneous implantable cardiac defibrillator (S-ICD) a valuable alternative in patients with adult congenital heart disease (ACHD). However, higher S-ICD ineligibility and higher inappropriate shock rates-mostly caused by T wave oversensing (TWO)- are observed in this population. We report a novel application of deep learning methods to screen patients for S-ICD eligibility over a longer period than conventional screening.

**Methods:**

Adult patients with ACHD and a control group of normal subjects were fitted with a 24-h Holters to record their S-ICD vectors. Their T:R ratio was analysed utilising phase space reconstruction matrices and a deep learning-based model to provide an in-depth description of the T: R variation plot for each vector. T: R variation was compared statistically using *t*-test.

**Results:**

13 patients (age 37.4 ± 7.89 years, 61.5 % male, 6 ACHD and 7 control subjects) were enrolled. A significant difference was observed in the mean and median T: R values between the two groups (p < 0.001). There was also a significant difference in the standard deviation of T: R between both groups (p = 0.04).

**Conclusions:**

T:R ratio, a main determinant for S-ICD eligibility, is significantly higher with more tendency to fluctuate in ACHD patients when compared to a population with normal hearts. We hypothesise that our novel model could be used to select S-ICD eligible patients by better characterisation of T:R ratio, reducing the risk of TWO and inappropriate shocks in the ACHD patient cohort.

## 1 Introduction

Sudden cardiac death (SCD) is a major cause of mortality in adult congenital heart disease (ACHD) patients, accounting for 19–26 % of deaths, the majority caused by ventricular arrhythmia [1]. The overall SCD incidence in ACHD patients is also higher than in the age-matched population without congenital heart disease [2]. As more patients with congenital heart disease survive into later life, rates of SCD are expected to rise as longer life expectancy increases the prevalence of arrhythmias owing to structural remodelling.

While the decision to implant ICD for secondary prevention is relatively straightforward, the decision to implant ICD for primary prevention in ACHD can be more challenging due to the lack of robust evidence in the ACHD population and is usually guided by the presence or absence of multiple risk factors: non sustained ventricular tachycardia (VT), impaired systemic ventricular function, inducible VT and syncope [1,3].

The subcutaneous implantable cardiac defibrillator (S-ICD) may especially be valuable for ACHD patients as potential anatomical challenges of transvenous lead implantation in ACHD patients can be overcome with a subcutaneous approach. Adult congenital heart disease patients are also younger and require several generator replacements during their lifetime increasing the risk of potential complications associated with TV-ICDs making them less appealing. Unfortunately, not all patients are eligible for S-ICD therapy. The eligibility for S-ICD is identified during a mandatory pre-implant screening process that is undertaken in all potential S-ICD recipients using guidelines by the device manufacturer. Surface ECG recordings taken in multiple body postures on the day of the screening are used as surrogates for S-ICD vectors to non-invasively assess vector morphology and determine S-ICD eligibility. Patients with an ECG morphology that does not meet the screening criteria are deemed to be at high risk of oversensing and inappropriate shocks and are subsequently deemed ineligible for an S-ICD. One major determinant of S-ICD eligibility is the T:R ratio. In fact, the most common cause of a vector failing S-ICD screening is a large T:R ratio [4,5].

Literature concerning the eligibility for S-ICD in patients with ACHD is scarce with only a handful of published studies with varying results. Alonso et al. conducted a study to test S-ICD eligibility specifically in ACHD patients at high risk of SCD and 69 (68 %) of Tetralogy of Fallot patients and 26 (80 %) of systemic RV patients were deemed eligible for a S-ICD [6]. In another study by Wang et al., 101 ACHD patients were screened and only 61 patients (60 %) passed the S-ICD screening [7]. Garside et al. also conducted prospective analysis on the S-ICD eligibility for 102 ACHD patients and 25 (24.5 %) patients failed the S-ICD screening criteria [8].

All the above-mentioned studies aside from the high variability of their outcomes demonstrated higher ineligibility rates in the ACHD population when compared with the general population. This may be due to abnormal T-wave morphology, resulting from the unique anatomical and physiological features that characterizes ACHD such as cardiac chamber enlargement, abnormal cardiac orientation, mechanical strain, and augmented repolarization patterns.

In the ﬁrst reported analysis comparing use of the S-ICD in patients with and without ACHD, D'Souza et al. conducted a pooled analysis of patients in the EFFORTLESS S-ICD registry and the U.S. IDE study. 19 patients with structural congenital heart disease were compared to 846 patients without ACHD. The overall complication rates were similar in the ACHD and non-ACHD groups (10.5 % vs. 9.6 %), with inappropriate shocks for T-wave oversensing being the only complication in the ACHD group; however, the rate of T-wave oversensing was higher (10.5 %) in ACHD patients when compared with non-ACHD patients (4.4 %). This analysis demonstrated that the S-ICD is a safe option for patients with ACHD deemed to be at high risk for SCD without having pacing indications [9].

In this study, we report a novel application of deep learning methods to screen ACHD patients for S-ICD eligibility over a longer period than conventional screening. We hypothesise that this screening approach might achieve better patient selection and optimise S-ICD vector selection in ACHD patients.

## 2 Methods

**Fig. 1:**
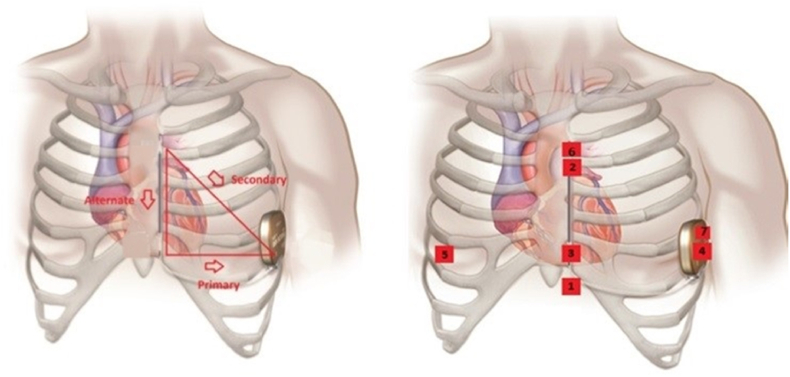
Showing the typical S-ICD vectors on the left and on the right, the Holter® surface ECG positions. 1 = 1 cm infero-lateral to the xiphisternum 2 = 14 cm superior to position 1 3 = 5th intercostal space, parasternal position 4 = 6th intercostal space left mid axillary line 6 = Adjacent to 2 7 = Adjacent to 4 Holter Channel A records between points 1 and 4 = surrogate of S-ICD primary vector Holter Channel B records between points 2 and 3 = surrogate of S-ICD alternate vector Holter Channel C records between points 6 and 7 = surrogate of S-ICD secondary vector 5 = 5th intercostal space right mid clavicular line = neutral electrode Image prior to annotation © Boston Scientific Corporation or its affiliates.

Healthy volunteers with structurally normal hearts and patients with a known diagnosis of ACHD were prospectively enrolled and asked to wear a seven lead/three channel Holter monitor for 24 h. Patients were advised to maintain normal activity during the recording period. The leads for the Holters were positioned so that they mimic and correspond to the three vectors (primary, alternate, and secondary) of an S-ICD, see Fig. 1.

The Holter monitors were not assessed for the presence of underlying arrhythmia episodes. If the participants required Holter monitoring for diagnostic purposes, this was left at the discretion of their clinical team as per usual standard clinical care.

Patients’ demographics were obtained from the medical records. There was no requirement for further patient follow up. The study was granted REC (17/SC/0623) and R&D (RHMCAR0528) approvals.

As the main cause of inappropriate shocks in the S-ICD population is T wave over-sensing (TWO), the T:R ratio was specifically monitored throughout the 24 h due to it being one of the main determinants of S-ICD eligibility. A T:R ratio eligibility cut-off of 1:3 was chosen based on the manual S-ICD screening tool following the manufacturer's guidelines; although the manual screening method is now largely superseded by automatic screening methods, they follow the same principles.

### 2.1 Utilisation of artificial intelligence in screening

Machine learning methods are already being used in the classification and the prediction of various cardiovascular diseases through ECG data analysis [[10], [11], [12], [13], [14], [15], [16], [17], [18]]. A well-recognized technique for processing ECG data is to create its phase space reconstruction matrix (PSR), a popular tool in waveform analysis for representing non-linear characteristics of time series data using delay maps.

The tool we developed is specifically designed to track and analyse the T:R ratios for the leads corresponding to the S-ICD vectors over the 24-h recordings. Raw data from the Holters were first downloaded in ASCII format at a frequency of 500 Hz (Hz), then were split into 10 s segments. Baseline drift correction techniques were then applied, followed by adaptive band stop filtering to suppress power-line noise with a frequency of 50 Hz while a low pass filter was used to remove the remaining high frequency noise. Then PSR was utilised to convert the ECG signal into 32x32 pixel PSR images, one image for each 10 s worth of ECG data. A deep learning model was trained to predict the T:R ratio from these PSR images with a high degree of accuracy. Our method diverges from standard approaches by using the whole PSR matrix as the input to this model, a technique which, to the best of our knowledge, has not been attempted before. The proposed method is capable of automatically extracting a set of features that are much more descriptive than those that are found manually with more time-consuming methods. This model is used to predict the T:R ratio for each 10-s segment of the 24-h recording from their corresponding PSR images. The end result is a plot showing the variation of the T:R ratios for each lead/S-ICD vector over the recorded period [19].

### 2.2 Tool validation

The deep learning tool was trained using 10-fold cross validation. ECG segments with pre-determined, manually measured T:R ratios were used to train the tool, while a proportion of the segments were blinded from the tool and were subsequently used for a series of experiments to assess the tool for accuracy. The outcome of the tool (predicted T:R values) was compared to the previously manually measured T:R values. Several standard accuracy parameters were used to assess the accuracy of the tool; Mean squared error (MSE) = 0.0122, Root mean squared error (RMSE) = 0.0938, and mean absolute error (MAE) = 0.046. Having an MAE of 0.046 means that on average the difference between the tool-predicted T:R ratio and the manually measured T:R was 0.046. The results of these accuracy parameters were very favourable denoting high level of accuracy for the tool. Dunn et al. have reported a detailed description of the creation of this model previously [20].

### 2.3 Statistical methods

Data analysis was done using RStudio 1.4.1106 running R 4.0.5. The distribution of the data was assessed using histograms, QQ plots and normality tests. The categorical data were represented as n/N (%) and continuous data as mean (SD). Welch two-sample *t*-test was used to compare between the two studied groups.

## 3 Results

13 patients were enrolled in the study. The mean age of the participants was 37.4 ± 7.89 years; there were 8 (61.5 %) males and 5(38.5 %) females. 6 of the participants were in the ACHD group (mean age 39 ± 16.36 years, 83.3 % male) and 7 (mean age 36 ± 6.11 years, 42.9 % male) in the control group of healthy volunteers. From the ACHD group, 2 had tricuspid atresia and Fontan's procedures, 1 had partial atrioventricular defect, 1 had double outlet right ventricle, dextrocardia and repaired ventricular septal defect, 1 had ventricular septal defect and patent ductus and 1 patient had common arterial trunk with previous complete repair. All the healthy volunteers did not have any underlying cardiac conditions, see Table 1 for patients' demographics.

**Table 1:** Patients' demographics.

Total Number of Participants		N = 13	ACHD group	Healthy Volunteers
			N = 6	N = 7
Demographics:	Mean age [years ± 95 % CI]	37.4 ± 7.89	39 ± 16.36	36 ± 6.11
	Male	8 (61.5 %)	5(83.3 %)	3(42.9 %)
Underlying cardiac anatomy:				
	Structurally normal heart		0	7
	Tricuspid atresia and Fontan's procedure		2	0
	Partial atrioventricular defect		1	0
	Double outlet right ventricle, dextrocardia and repaired ventricular septal defect		1	0
	Ventricular septal defect and patent ductus		1	0
	Common arterial trunk with previous complete repair		1	0

**Fig. 2:**
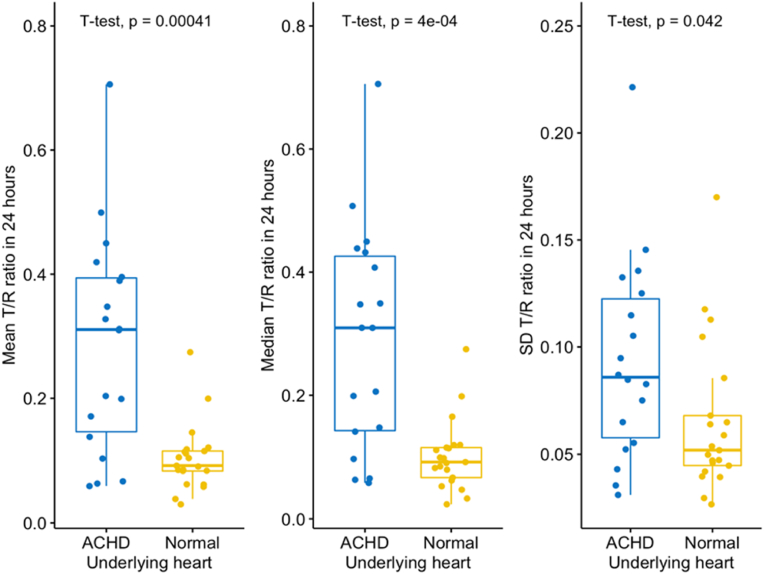
Mean, median, and SD of the T:R ratios measured in 24 h for the all the Leads/S-ICD vectors in ACHD and healthy volunteers with normal hearts groups.

When the results from all the leads/S-ICD vectors were combined, there was a statistically significant difference in the mean, median and the standard deviation (SD) of the T:R ratios measured in 24 h between both groups. The mean T:R ratio was higher in the ACHD (0.29 ± 0.18 versus 0.1 ± 0.05, p < 0.001). The median T: R was higher in the ACHD group (0.29 ± 0.18 versus 0.1 ± 0.06, p < 0.001) and the SD of the T:R ratio was also higher in the ACHD group (0.09 ± 0.05 versus 0.06 ± 0.04, p = 0.042). in other words, the T:R ratio was higher and exhibited more tendency to fluctuate (SD) in the ACHD group when compared to the healthy volunteers, see Table 2 and Fig. 2.

**Table 2:** Mean, median, and SD of the T:R ratios measured in 24 h for the all the Leads/S-ICD vectors.

Parameters	N	Underlying Anatomy		p-value^a^
		**ACHD**, N = 18	**Normal**, N = 21	
Mean T: R ratio	39	0.29 (0.18)	0.10 (0.05)	**<0.001**
SD of T:R ratio	39	0.09 (0.05)	0.06 (0.04)	**0.042**
Median T: R ratio	39	0.29 (0.18)	0.10 (0.06)	**<0.001**

N = total number of the Leads/S-ICD vectors. ACHD = adult congenital heart disease. SD = standard deviation. IQR = interquartile range.

a Welch Two Sample *t*-test.

**Fig. 3:**
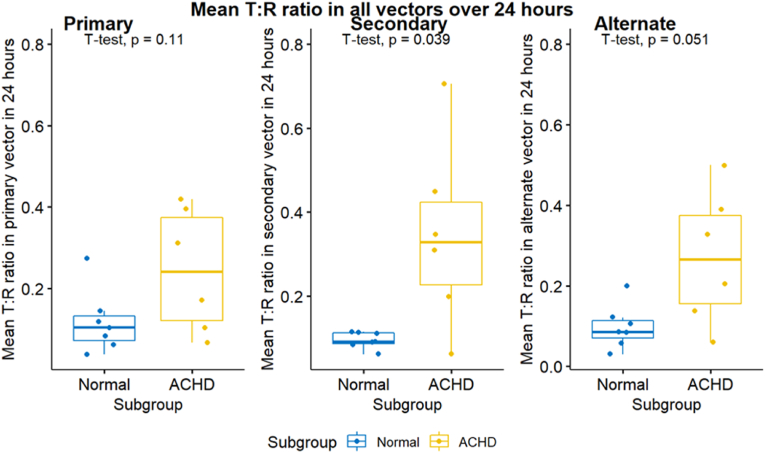
Mean T:R ratios measured in 24 h classified according to the S-ICD vector.

**Fig. 4:**
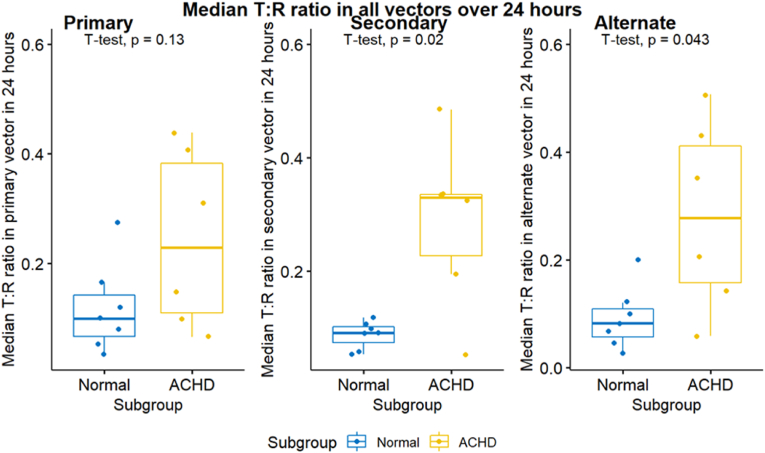
Median T:R ratios measured in 24 h classified according to the S-ICD vector.

**Fig. 5:**
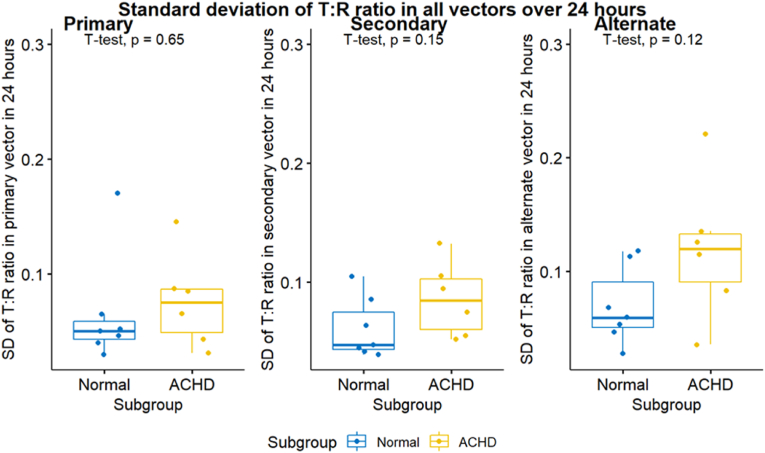
Standard deviation (SD) of the T:R ratios measured in 24 h classified according to the S-ICD vector.

T:R ratios were also assessed in each of the three S-ICD vectors separately. Mean T:R ratios were higher in the ACHD group in all vectors; (0.245 versus 0.118, p = 0.11) in the primary vector, (0.346 versus 0.096, p = 0.039) in the secondary vector and (0.269 versus 0.097, p = 0.051) in the alternate vector. Median T: R ratios were higher in the ACHD in all vectors; (0.244 versus 0.118, p = 0.13) in the primary vector, (0.288 versus 0.088, p = 0.02) in the secondary vector and (0.282 versus 0.091, p = 0.043) in the alternate vector. The SD of the T: R ratios were also higher in the ACHD for all vectors; (0.076 versus 0.065, p = 0.65) for the primary vector, (0.086 versus 0.061, p = 0.15) for the secondary vector and (0.119 versus 0.069, p = 0.12) for the alternate vector. This means that, for the ACHD group, the secondary vector had the highest T:R ratio (least favourable from S-ICD perspective) at the baseline, followed by the alternate vector then the primary vector. However, T:R ratio demonstrated highest degree of fluctuations in the alternate vector followed by the secondary then the primary vectors in the ACHD group. Differently, for the healthy volunteers’ group, the primary vector had the highest T: R ratio at the baseline and all the vectors exhibited the same degree of T: R ratio fluctuations, see Table 3 and Fig. 3, Fig. 4, Fig. 5.

**Table 3:** Mean, median, and SD of the T:R ratios measured in 24 h classified according to the S-ICD vector.

Parameters	N	ACHD (N = 6)	Normal (N = 7)	P-Value	95 % CI
Mean T: R Pr. vector	13	0.245	0.118	0.105	(-0.288,0.034)
Mean T: R S. vector	13	0.346	0.096	0.039	(-0.481, −0.019)
Mean T: R Alt. vector	13	0.269	0.097	0.051	(-0.345,0.001)
Median T: R Pr. Vector	13	0.244	0.118	0.126	(-0.299,0.046)
Median T: R S. vector	13	0.288	0.088	0.021	(-0.354, −0.045)
Median T: R Alt. vector	13	0.282	0.091	0.043	(-0.375, −0.008)
SD T: R Pr. Vector	13	0.076	0.065	0.649	(-0.065,0.043)
SD T: R S. vector	13	0.086	0.061	0.15	(-0.060,0.011)
SD T: R Alt. vector	13	0.119	0.069	0.119	(-0.116,0.016)

N= Number of patients. Pr. Vector, S. vector, and Alt. vector = Primary, secondary, and alternate vectors respectively.

Our tool is designed to give the T:R ratio for every 10 s of data/ECG signals – equivalent to a standard 12-lead ECG or a standard ECG strip used for current S-ICD screening process [21] - this allowed the assessment of every individual lead/S-ICD vector eligibility for every 10 s for the whole 24-h screening. From there, the probability of each vector failing the current screening methodology if the screenings were done at any time of the day was calculated. Where the probability of failure = number of 10-s segments with unfavourable (>1:3) T:R ratio/total number of 10-s segments (8640) in a 24-h recording.

The probability of an S-ICD vector failing the screening for our ACHD cohort averaged at 0.36 ± 0.31, 0.36 ± 0.28, and 0.38 ± 0.25 for the primary, alternate, and secondary vectors, respectively. While the probability of a vector failing the screening in our healthy volunteers’ group was significantly lower at 0.04 ± 0.08, 0.03 ± 0.04, and <0.01 for the primary, alternate, and secondary vectors respectively, see Table 4.

**Table 4:** Shows the probability of S-ICD screening failure for all the S-ICD vectors in the study.

ID	Group	Primary Vector		Alternate Vector		Secondary Vector	
		10-s segments of T: R > 1/3	Probability of failing screening	10-s segments >1/3	Probability of failing screening	10-s segments >1/3	Probability of failing screening
1	ACHD	2450	0.28	1235	0.14	4364	0.51
2	ACHD	7106	0.82	6077	0.7	6998	0.81
3	ACHD	0	0	4800	0.55	4538	0.52
4	ACHD	7488	0.87	6573	0.76	3733	0.43
5	ACHD	1411	0.16	34	<0.01	35	<0.01
6	ACHD	0	0	1	<0.01	122	0.01

	**Mean**	3756 ± 2720	0.36 ± 0.31	3120 ± 2435	0.36 ± 0.28	3298 ± 2185	0.38 ± 0.25

7	Normal	3	<0.01	0	0	23	<0.01
8	Normal	2613	0.3	19	<0.01	0	0
9	Normal	17	<0.01	51	<0.01	0	0
10	Normal	1	<0.01	3	<0.01	1	<0.01
11	Normal	0	0	452	0.05	14	<0.01
12	Normal	2	<0.01	12	<0.01	277	0.03
13	Normal	1	<0.01	1074	0.12	3	<0.01

	**Mean**	377 ± 731	0.04 ± 0.08	230 ± 301	0.03 ± 0.04	45 ± 76	<0.01

## 4 Discussion

The concept of the potential varying of S-ICD vectors eligibility over time was introduced before in a published study by Wiles et al. The study has demonstrated that the vector score, which determines S-ICD eligibility, is in fact dynamic in real-life ICD population [22]. The clinical significance for this dynamicity is not clear but it sheds the light on the possibility that acquiring screening data over a much longer period than for conventional screening across the three S-ICD vectors can enable more reliable and descriptive screening of the vectors and can aid patient and vector selection in S-ICD candidates.

We reported a novel application of artificial intelligence and deep learning methods used to screen patients for S-ICD eligibility. Screening data was acquired over a much longer period than for conventional screening approaches and provides an in-depth description of the behaviour of the T:R ratio over that period across the S-ICD vectors [19].

This novel approach allowed the detailed description of the behaviour of the T: R ratio over prolonged screening periods. This has demonstrated that one of the main determinants of S-ICD eligibility – the T: R ratio – is dynamic. Upon analysing the results down to the individual level, the clinical significance became apparent. These small changes in the T:R ratio parameters can dictate the S-ICD eligibility as dynamic changes in the T:R ratio in some of the vectors that were observed were significant enough in some instances to cause the T:R ratio to cross the threshold for the S-ICD screening. It comes as a no surprise that the probability of S-ICD vectors failing the screening in the ACHD group was much higher than that of the healthy volunteers with structurally normal hearts in our study, which is in line with previously published [[6], [7], [8]] This may be due to abnormal T-wave morphology, resulting from the unique anatomical and physiological features that characterizes ACHD such as cardiac chamber enlargement, abnormal cardiac orientation, mechanical strain and augmented repolarization patterns.

This novel screening approach could enable more reliable assessment of ACHD patients’ eligibility for S-ICD implantation and guide patient selection for S-ICD therapy with lower risk of inappropriate shock therapy due to TWO. This is important as inappropriate shock therapies can have detrimental effects on the quality of life, psychological wellbeing and can even result in the induction of ventricular arrhythmias [23].

It is not uncommon for multiple vectors to pass the S-ICD screening. In current practice, the choice of which vector to use for programming is arbitrary since the outcome of the screening is binary (pass or fail) and there are no “degrees” awarded for an S-ICD vector for passing the screening. Our tool can guide the selection of the most favourable vector for programming the S-ICD. The most favourable vector would be the most stable or the one that is least likely to fluctuate and cross the screening threshold during prolonged screening and thus pose the least risk of TWO and inappropriate shocks.

ACHD is a broad term covering a wide array of underlying anatomical variants. It is as such expected that there would be a high degree of variability not only of the S-ICD screening passing rate, but also of which of the S-ICD vectors that are likely to pass the screening in the ACHD population. It is prudent that vector selection for S-ICD programming should be individualised for each patient and our novel screening approach could enable more reliable and descriptive assessment of the S-ICD vectors behaviour over prolonged screening periods allowing clinicians to make more informed decisions on vectors selection in S-ICD eligible patients. This can be translated into lower risk of TWO and inappropriate shock therapy.

The cut-off T:R ratio of 1:3 used for current screening practice incorporates a safety margin to accommodate for the fluctuations of the ECG signal amplitudes over time without affecting the sensing of the S-ICD. Currently, patients who do not possess at least a single S-ICD vector meeting this T:R ratio cut-off are deemed ineligible for S-ICD therapy, a significant limitation to their care, particularly in ACHD patients where S-ICD provides a valuable and, in some cases, their only option for defibrillation protection therapy. Our screening approach using our proposed tool can accurately measure the degree of the T:R ratio fluctuation over the screening period potentially eliminating the need to incorporate a “safety-margin” into the eligibility threshold of the T:R ratio. Clinically, this can be translated into higher rates of S-ICD eligibility without having to compromise with a higher risk of TWO and inappropriate shocks. Further prospective studies with real-life S-ICD candidates and long-term follow up will be needed to give insight into the optimal T:R ratio that could be utilised for prolonged screening for S-ICD eligibility.

### 4.1 Limitations

There are several limitations to our study. The number the patients recruited in this study is small and ACHD is treated as a single entity for the sake of our analysis when in reality ACHD covers very different underlying pathologies with various degrees of complexities and larger adequately powered studies are needed to consolidate our findings. Second, none of the patients recruited in our study had an indication for a S-ICD. In addition, while participants were advised to maintain normal activity during the recording period of 24 h, to try to mimic an average day of a potential S-ICD recipient. Participants were not asked to keep a diary of their activities/postures at a given time or their sleeping schedule on that day which could provide significant insights on the postural as well as the diurnal variation in the T:R ratios. We recommend that this should be considered in further work.

Also, our study focuses on the T:R ratio as the major determinant of S-ICD eligibility, not counting any other parameters which can contribute to the passing or failing of the S-ICD screening. Furthermore, while a statistical analysis comparing our tool outcomes against manual measurement of the T:R would have been of great value. It was not practically feasible to manually measure the T:R ratios for the recordings due to the sheer magnitude of the data collected for our study (>3 million QRS complexes).

Our proposed methodology also does not consider relatively newer algorithms such as SMART PASS that are integrated into the S-ICD that can help it differentiate between R and T waves based on other characteristics rather than just their amplitudes. Finally, the clinical relevance of our findings is not very evident; while we have demonstrated that there is a variation of T:R ratio in ACHD patients, there is no evidence that this would inadvertently lead to TWO or inappropriate shocks. Theoretically, our tool could be potentially used to predict the risk of TWO events and allow informed decisions to be made by the physicians and patients alike prior to committing to S-ICD therapy. However, further work is needed before it is possible to apply our tool to clinical practice.

## 5 Conclusions

T:R ratio, one of the major determinants for S-ICD eligibility, is significantly higher and exhibited more tendency to fluctuate in ACHD patients when compared to normal hearts populations. Our novel model utilises artificial intelligence and deep learning methods to provide an in-depth description of the behaviour of the T:R ratio across the S-ICD vectors. This could be used to better assess S-ICD eligibility and guide S-ICD vector selection, thus reducing the risk of TWO and inappropriate shocks particularly in the ACHD patients' cohort.

## Funding

None.

## Declaration of competing interest

x The authors declare the following financial interests/personal relationships which may be considered as potential competing interests:

-Dr. Mohamed ElRefai has received an unrestricted grant from Boston Scientific.

-Dr. Benedict Wiles has received unrestricted research funding and consultancy payments from Boston Scientific.

-Dr. Paul Roberts receives consultancy fees from Boston Scientific and Medtronic.

-Professor John Morgan is a medical director at Boston Scientific.

- None of the other authors of this study has conflict of interest to declare.
